# Factors influencing stigma in Chinese postoperative breast cancer patients: a systematic review and meta-analysis

**DOI:** 10.3389/fmed.2025.1681487

**Published:** 2025-10-09

**Authors:** Wenjuan Yan, Hongjuan Zhou, Yinying Huang, Yunfei Zhang, Shaofang Wang, Xuefen Yu

**Affiliations:** ^1^Department of Nursing, Women and Children’s Hospital, School of Medicine, Xiamen University, Xiamen, Fujian, China; ^2^School of Nursing, Li Ka Shing Faculty of Medicine, University of Hong Kong, Hong Kong, Hong Kong SAR, China; ^3^Department of Interventional Radiology Center, School of Medicine, Xiamen Cardiovascular Hospital of Xiamen University, Fujian Branch of National Clinical Research Center for Cardiovascular Diseases, Xiamen, Fujian, China; ^4^Department of Breast Diseases, Women and Children’s Hospital, School of Medicine, Xiamen University, Xiamen, Fujian, China; ^5^Department of Obstetrics and Gynecology, Women and Children’s Hospital, School of Medicine, Xiamen University, Xiamen, Fujian, China

**Keywords:** breast neoplasms, social stigma, risk factors, Chinese patients, systematic review, meta-analysis

## Abstract

**Objective:**

To systematically evaluate stigma levels and its influencing factors among Chinese postoperative breast cancer patients, providing evidence for culturally adapted interventions.

**Methods:**

Chinese and English databases, such as China Biology Medicine Disc (CBM), China National Knowledge Infrastructure (CNKI), Wanfang Data Knowledge Service Platform (WangFang), China Science and Technology Journal Database (The VIP), Cochrane Library, PubMed, Embase, Scopus, Medline, Web of Science, and grey literature from the National Institute for Health Research (NIHR), were searched. The time limit of the search for literature on the factors affecting the level of stigma in Chinese postoperative breast cancer patients was from the establishment of the databases to 14 July 2025. Two researchers independently screened studies, extracted data, and assessed quality using AHRQ criteria. Meta-analyses were performed using Stata 18.0.

**Results:**

Fourteen cross-sectional studies (total *n* = 2,873 patients) revealed a high stigma burden among Chinese postoperative breast cancer patients, with pooled Social Impact Scale (SIS) scores of 58.22 (95%*CI*: 55.30—61.15)—significantly exceeding rates in comparable populations. Meta-analysis identified seven culturally embedded predictors (*p* < 0.05): higher stigma was associated with unmarried/divorced/widowed status (*SMD* = 1.754), rural residence (*SMD* = 1.337), negative body image (*SMD* = 0.467), and yielding coping styles (*SMD* = 1.276); conversely, lower stigma correlated with occupational engagement (*SMD* = −0.568), breast reconstruction (*SMD* = −2.116), and self-payment status (*SMD* = −0.747). Paradoxically, spousal support intensified stigma (*β* = 1.336), while broader social support showed no significant association (*p* = 0.680).

**Conclusion:**

Chinese breast cancer survivors face severe stigma shaped by Confucian familial norms, economic pressures, and healthcare disparities. Interventions must prioritize marital counseling, occupational reintegration, accessible reconstruction, and rural mental health services. Longitudinal studies are needed to establish causality.

**Systematic review registration:**

CRD42024502898, https://www.crd.york.ac.uk/PROSPERO/recorddashboard.

## 1 Introduction

Breast cancer, as the most prevalent malignant tumor among Chinese women, has seen its disease burden continuously exacerbated by population aging and lifestyle changes (1). The 2022 National Cancer Report indicates that China reports approximately 357,200 new breast cancer cases annually, with projections suggesting this number will exceed 500,000 by 2030 (2). A younger onset trend (peak incidence: 45–54 years) and high proportion of late-stage diagnoses (>30%) constitute distinctive national characteristics (3, 4). Although China’s age-standardized incidence rate (33.0/100,000) remains lower than Western countries, the annual increase rate over the past decade reached 3.9%, ranking among the highest globally (5, 6). More notably, while the 5-year relative survival rate for Chinese patients has significantly improved to 83% (7, 8), quality-of-life issues due to physiological impairment and psychological trauma are increasingly prominent (9). Currently, surgery remains the cornerstone of treatment for localized breast cancer (10), with the vast majority of patients undergoing surgical intervention. Therefore, understanding the psychosocial experiences of this specific postoperative population is critical.

China’s unique sociocultural context shapes the experience of stigma among breast cancer patients in distinct ways (11). Influenced by traditional gender-role expectations, breast loss is often perceived as a deficiency in female identity, which may lead patients to face a three-fold dilemma: marital crisis, workplace discrimination, and social isolation (12, 13). Notably, one study reported that 76.7% of Chinese patients conceal their condition due to fear of discrimination (14), which highlights a significant “burden of silence” within this population. This concealment can directly reduce treatment adherence and double the risk of depression. Current breast cancer research in China reveals two major gaps: First, insufficient localization of influencing factors. Existing international stigma prediction models (e.g., body image, social support) inadequately consider China-specific familial power structures and urban–rural healthcare disparities (15). Second, contradictory empirical conclusions: While some studies show no significant correlation between marital status and stigma (16), others indicate that being unmarried/divorced/widowed is an independent influencing factor for stigma in postoperative breast cancer patients, with stigma scores 5.425 times higher than married individuals (*p* < 0.05) (17, 18). These discrepancies highlight the moderating effect of cultural variables.

Therefore, this study will focus exclusively on the Chinese population for the first time, aiming to integrate evidence on stigma influencing factors in mainland China through systematic review and meta-analysis, to provide an evidence base for analysing how healthcare system characteristics regulate stigma and constructing China-specific intervention strategies.

## 2 Methods

### 2.1 Registration and protocol

This review has been registered with the International Prospective Register of Systematic Reviews (PROSPERO), and the registered number is CRD42024502898. This systematic review was conducted and reported in accordance with the Preferred Reporting Items for Systematic Reviews and Meta-Analyses (PRISMA) 2020 statement (19).

### 2.2 Search strategy

The Chinese and English databases searched were as follows: (a) the Chinese databases included China Biology Medicine Disc (CBM), China National Knowledge Infrastructure (CNKI), Wanfang Data Knowledge Service Platform (WangFang), and China Science and Technology Journal Database (The VIP); (b) the English databases included Cochrane Library, PubMed, Embase, Scopus, Medline, and Web of Science; and (c) grey databases were also searched, including the National Institute for Health Research (NIHR). The search included all observational studies that described the level of stigma and factors influencing it in postoperative breast cancer patients. The search period was from the establishment of each database to 14 July 2025. Table 1 shows an example of one of the databases searched, namely, PubMed. The search formula and related information are detailed in Supplementary material 1.

**Table 1:** Search strategy in PubMed.

Step (#)	Search Strategy
#1	“Breast Neoplasms” [MeSH Terms]
#2	“Breast Neoplasm” OR “Neoplasm, Breast” OR “Breast Tumors” OR “Breast Tumor” OR “Tumor, Breast” OR “Tumors, Breast” OR “Neoplasms, Breast” OR “Breast Cancer” OR “Cancer, Breast” OR “Mammary Cancer” OR “Cancer, Mammary” OR “Cancers, Mammary” OR “Mammary Cancers” OR “Malignant Neoplasm of Breast” OR “Breast Malignant Neoplasm” OR “Breast Malignant Neoplasms” OR “Malignant Tumor of Breast” OR “Breast Malignant Tumor” OR “Breast Malignant Tumors” OR “Cancer of Breast” OR “Cancer of the Breast” OR “Mammary Carcinoma, Human” OR “Carcinoma, Human Mammary” OR “Carcinomas, Human Mammary” OR “Human Mammary Carcinomas” OR “Mammary Carcinomas, Human” OR “Human Mammary Carcinoma” OR “Mammary Neoplasms, Human” OR “Human Mammary Neoplasm” OR “Human Mammary Neoplasms” OR “Neoplasm, Human Mammary” OR “Neoplasms, Human Mammary” OR “Mammary Neoplasm, Human” OR “Breast Carcinoma” OR “Breast Carcinomas” OR “Carcinoma, Breast” OR “Carcinomas, Breast” [Title/Abstract]
#3	#1 OR #2
#4	“Social Stigma” [MeSH Terms]
#5	“Social Stigmas” OR “Stigmas, Social” OR “Stigma, Social” OR “Stigma” OR “internalized stigma” OR “externalized stigma” OR “selfstigma” OR “shame” [Title/Abstract]
#6	#4 OR #5
#7	“influence factors” OR “related factors” OR “risk factors” OR “predictive factors” OR “current situation” OR “investigation” OR “relevance” [Title/Abstract]
#8	#3 AND #6 AND #7

### 2.3 Eligibility criteria

#### 2.3.1 Inclusion criteria

1. Study design: Observational studies (case–control, cohort, cross-sectional) reporting current stigma status and its influencing factors in Chinese postoperative breast cancer patients.
2. Participants: Chinese breast cancer patients (≥18 years old) with histologically confirmed diagnosis who underwent surgical treatment.
3. Outcome measures:

- Report the level of stigma and include the application of at least one stigma-related scales
- Report results in Linear regression models: Providing standardized regression coefficients (*β*) with SEs or complete data for SMD calculation (means, SDs, sample sizes)

(4) Predictors: Report ≥2 statistically quantified predictors of stigma.
(5) Data availability: Sufficient data including Means ± SDs or regression coefficients with SEs.
(6) Languages: Publications in Chinese or English.
(7) Geographic scope: Studies conducted in mainland China.

#### 2.3.2 Exclusion criteria

1. Duplicate publications from the same author/research group.
2. Journal articles duplicating dissertation content.
3. Unrecoverable incomplete data (after contacting authors).
4. Studies reporting only significance levels (*p*-values) without quantitative effect measures.
5. Studies using non-validated or non-standard stigma assessments.

### 2.4 Literature screening and data extraction

All potentially relevant studies were imported into Covidence for the purpose of eliminating duplicate studies and subsequent screening. Two reviewers (Yan WJ and Zhou HJ) independently conducted a comprehensive literature search and imported the relevant articles into the literature management software EndNote. The inclusion and exclusion criteria were strictly followed to determine the selection of the literature, which was cross-checked for accuracy. Any disagreements between the researchers were resolved through discussion, with consultation from a third researcher (Zhang YF) when necessary. Additionally, data extraction was performed separately by two researchers (Yan WJ and Zhou HJ), including information such as authors, year of publication, country of origin, study design, stigma identification methods, sample size, stigma scores, and influential factors.

### 2.5 Literature quality evaluation

The included literature was independently evaluated by two researchers (Yan WJ and Zhou HJ) according to the Agency for Healthcare Research and Quality’s (AHRQ) criteria for assessing the risk of bias in observational studies (20). The evaluation consisted of 11 entries, with a score of 0 to 3 indicating low quality, 4 to 7 moderate quality, and 8 to 11 high quality. After completing the quality evaluation, the results were discussed and negotiated, and the quality of the included literature was categorized as high quality (Grade A), moderate quality (Grade B), and low quality (Grade C). Grade C literature was excluded.

### 2.6 Data synthesis and statistical analysis

Statistical analyses were performed using Stata 18.0 software. The analytical approach comprised two distinct phases addressing separate research objectives.

#### 2.6.1 Phase 1: meta-analysis of stigma scores

A single-arm synthesis was conducted to estimate pooled mean stigma scores. Raw mean values with corresponding standard deviations (or standard errors) served as the primary data for synthesis. The DerSimonian-Laird random-effects model was applied to account for anticipated heterogeneity across studies. The use of means and standard deviations for meta-analysis is conventional for continuous outcome data and assumes that the stigma scores from the included studies approximate a normal distribution and can be treated as parametric data. Subgroup analyses were stratified by measurement scale type to examine instrument-specific effects. Results were expressed as weighted mean values with 95% confidence intervals.

#### 2.6.2 Phase 2: meta-analysis of influencing factors

For factors reported in <2 studies, descriptive synthesis was performed by reporting individual study effect sizes without quantitative pooling. For factors reported in ≥2 studies, standardized mean differences (SMD) were computed as the comparative effect measure. All factors were analyzed using inverse-variance weighted fixed-effects or random-effects models, selected based on the results of heterogeneity tests.

#### 2.6.3 Heterogeneity assessment

Between-study heterogeneity was quantified using the *I*^2^ statistic, with statistical significance assessed via Cochran’s Q-test employing an *α*-threshold of 0.10. Model selection adhered to predefined criteria whereby random-effects models were applied when *I*^2^ ≥ 50% or Q-test *p*-value ≤0.10, while fixed-effects models were utilized when *I*^2^ < 50% and Q-test *p*-value >0.10.

#### 2.6.4 Sensitivity analysis

To evaluate result robustness, we performed model consistency comparisons between fixed and random effects estimates, supplemented by sequential exclusion procedures involving iterative removal of individual studies. For factors where sensitivity analysis indicated instability and substantial heterogeneity (*I*^2^ ≥ 50%), we further employed Baujat plots to objectively identify and visualize outlying studies that exerted a disproportionate influence on both the pooled effect size and the between-study heterogeneity. This method provides a quantitative and graphical assessment of influence, complementing the sequential exclusion approach. All influence diagnostics were conducted using the R software (version 4.5.1).

#### 2.6.5 Publication bias assessment

Potential publication bias was assessed using visual funnel plot inspection applied to factors with ≥10 studies, where *p* > 0.10 indicated no significant bias.

## 3 Results

### 3.1 Study selection

**Figure 1:**
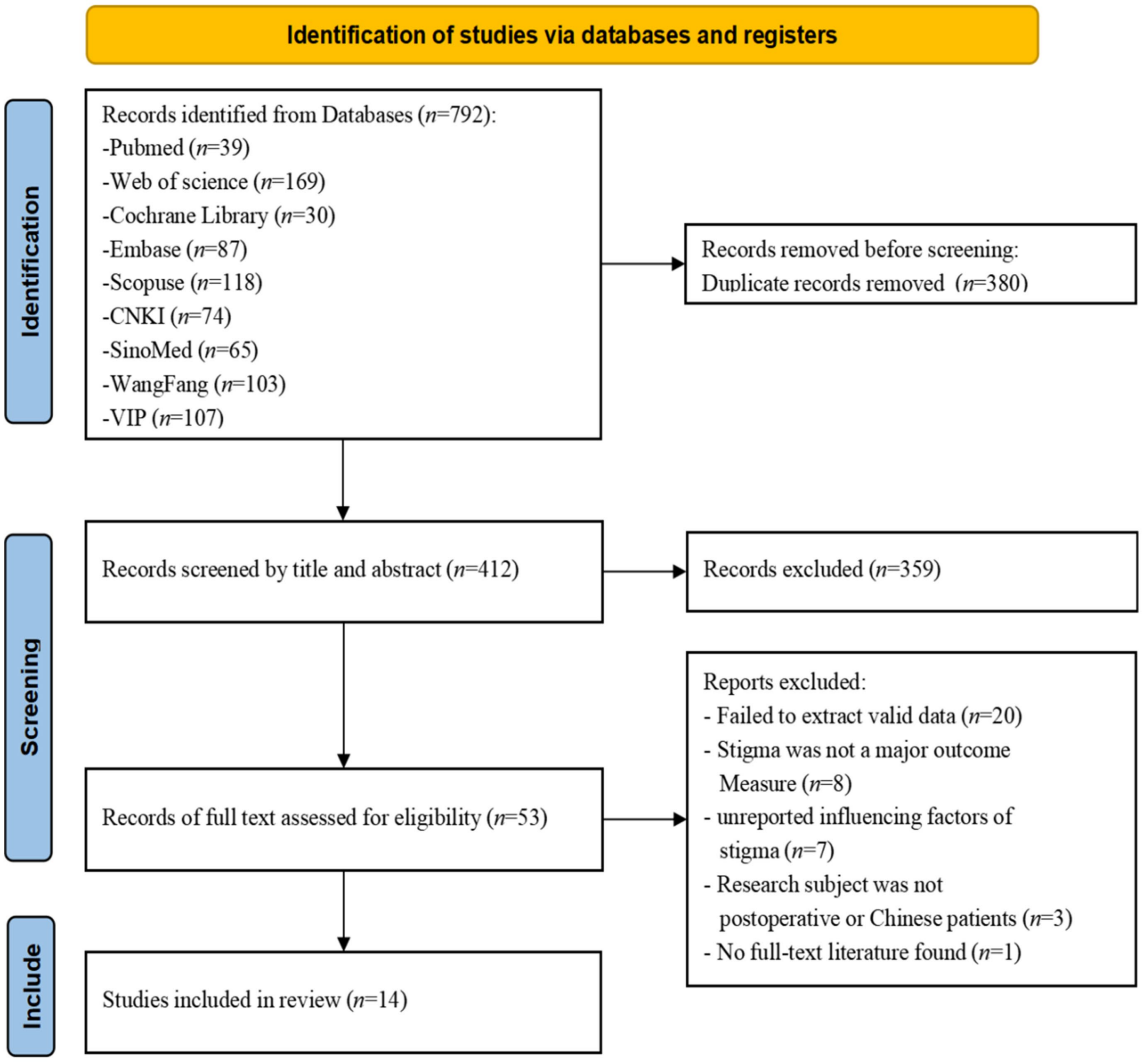
PRISMA flow diagram of the study selection process.

A total of 792 potentially eligible literature articles were extracted from various databases, of which 380 were removed based on duplicated records. The remaining 412 articles were checked manually by Covidence and EndNote software; 359 articles were eliminated after reading the titles and abstracts, and 39 articles were eliminated after reading the entire texts. Ultimately, 14 studies with a total of 2,873 patients satisfied the inclusion requirements (see Figure 1). The excluded studies and reasons are detailed in Supplementary material 2.

### 3.2 Basic information and methodological quality assessment of the included studies

The 14 included studies were cross-sectional studies, and in terms of tools for evaluating patients’ stigma, 12 studies (18, 21–31) used the Social Impact Scale (SIS), two studies (32, 33) used the Link Stigma Series Scale (Link). Quality evaluation was performed according to the AHRQ’s recommended criteria for evaluating the risk of bias regarding observational studies. This resulted in 14 included studies that were all Grade A and Grade B literature. The details are shown in Table 2. The original data of the article are detailed in Supplementary material 3.

**Table 2:** Basic information of included studies and methodological quality evaluation results.

Author	Year	Country	Area	Study design	Stigma identification	Sample size	Scores of stigma	Influence factors	Quality assessment scores and levels
SHI JQ (18)	2018	China	Zhejiang	Cross-sectional	SIS	186	61.89 ± 8.41	①②③	10A
HU LL (21)	2022	China	Hubei	Cross-sectional	SIS	240	61.04 ± 11.99	②③④⑤	7B
WEI Y (22)	2020	China	Jiangsu	Cross-sectional	SIS	82	61.05 ± 11.98	①⑥	9A
QIN ZW (23)	2021	China	Zhejiang	Cross-sectional	SIS	368	66.54 ± 14.81	⑦	9A
AO YT (24)	2023	China	Jiangxi	Cross-sectional	SIS	82	57.32 ± 8.52	①④⑧⑨	6B
ZHENG CR (25)	2018	China	Guangdong	Cross-sectional	SIS	320	56.19 ± 10.11	①②⑤⑩	7B
XIE XW (26)	2020	China	Jilin	Cross-sectional	SIS	266	57.67 ± 6.00	⑦⑨⑩⑪⑫	9A
HE DM (27)	2020	China	Sichuan	Cross-sectional	SIS	255	62.38 ± 9.37	①③④⑤⑩⑫⑬	9A
JIANG F (32)	2022	China	Henan	Cross-sectional	Link	92	118.92 ± 10.76	②④⑥⑧⑨⑭⑮	9A
LI X (33)	2023	China	Jilin	Cross-sectional	Link	280	102.9 ± 5.43	①④⑤⑯	9A
LU F (28)	2025	China	Henan	Cross-sectional	SIS	86	63.17 ± 10.57	④⑥⑧⑭⑰	8A
PENG XY (29)	2025	China	Zhejiang	Cross-sectional	SIS	102	50.35 ± 4.69	①②④⑧	5B
WANG FJ (30)	2024	China	Gansu	Cross-sectional	SIS	242	49.07 ± 20.56	①②⑫⑬⑮	9A
WANG JH (31)	2024	China	Beijing	Cross-sectional	SIS	272	51.56 ± 11.74	⑱	9A

① age; ② monthly income; ③ work status; ④ education; ⑤ postoperative time; ⑥ coping style; ⑦ body image; ⑧ marital status; ⑨ social support; ⑩ surgery type; ⑪ spouse attitude; ⑫ occupation; ⑬ payment; ⑭ breast reconstruction; ⑮ residence; ⑯ negative emotion; ⑰ family support; ⑱ self-efficacy.

### 3.3 Meta-analysis results

#### 3.3.1 Status of stigma in postoperative breast cancer patients

**Figure 2:**
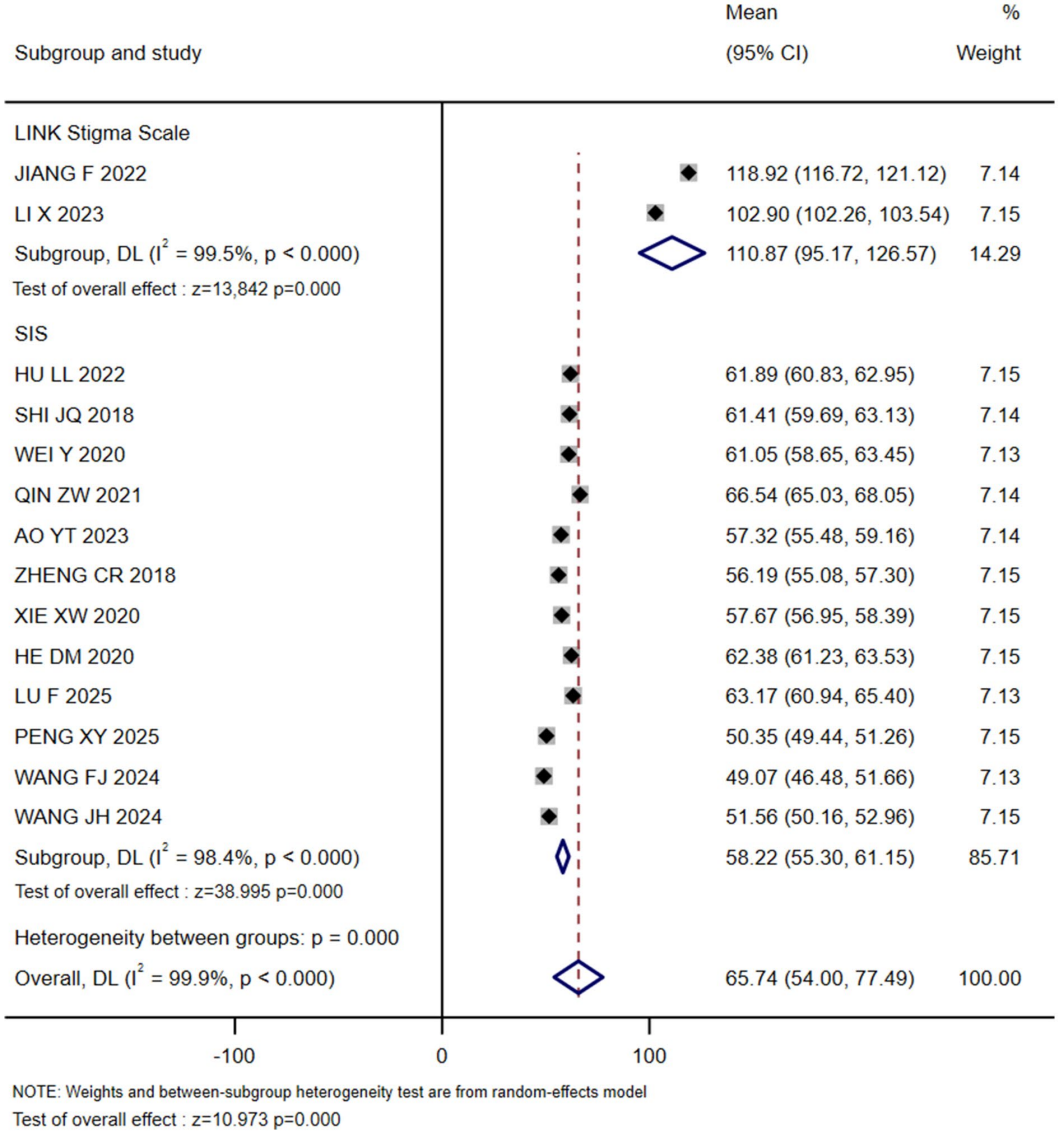
Forest map of the current situation of stigma in breast cancer patients after surgery.

12 studies used the SIS (18, 21–31), and the subgroup concordance results showed a stigma score of 58.22 (55.30, 61.15); and two studies used the Link Stigma Series Scale (32, 33), and the subgroup concordance results showed a stigma score of 110.87 (95.17, 126.57) (see Figure 2). The results of all 14 studies indicated that stigma was prevalent to varying degrees in postoperative breast cancer patients.

#### 3.3.2 Influencing factors of stigma in postoperative breast cancer patients

##### 3.3.2.1 Descriptive statistics

Eighteen potential influencing factors were identified across the 14 included studies. The following influencing factors were each reported by a single study: XIE XW et al. (26) found that spouse attitude was an independent predictor of stigma (*β* = 1.336, *p* < 0.01). Notably, when the spouse attitude was “caring,” the level of stigma significantly increased. Concurrently, the study by LI X et al. (33) reported a strong positive correlation between the total score of negative emotions and the sense of stigma (*r* = 0.530, *p* < 0.01). On the other hand, protective factors were also shown to effectively reduce these feelings. LU F et al. (28) found that good family support could enhance patients’ courage and confidence in facing external pressure, thereby reducing postoperative stigma (*β* = 0.277, *p* < 0.001). Similarly, WANG JH et al. (30) confirmed that the total score of self-compassion was significantly negatively correlated with stigma (*r* = −0.349, *p* < 0.01), which can independently explain 12.2% of the variation in stigma (*R^2^* = 0.122, *p* < 0.001).

##### 3.3.2.2 Meta-analysis findings

**Figure 3:**
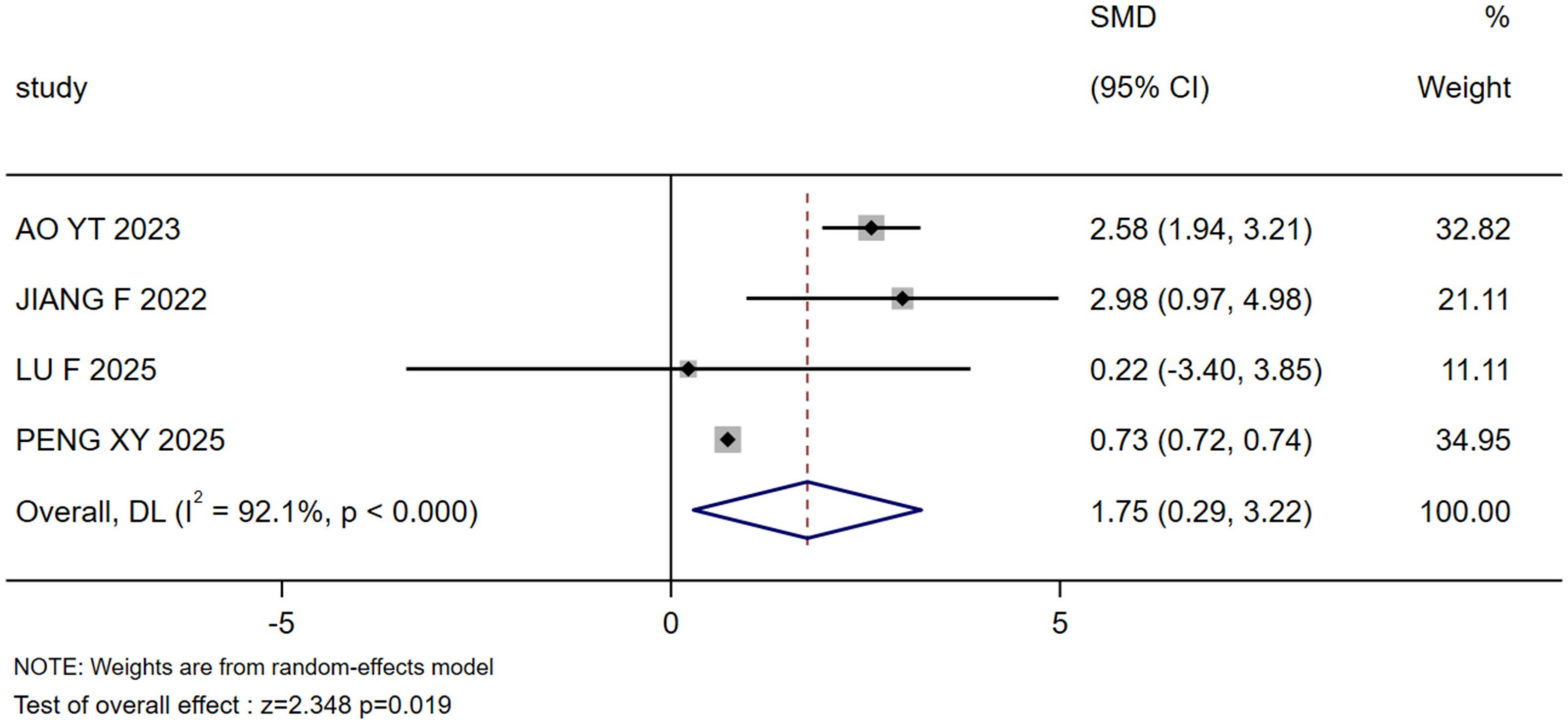
Forest plot: association between marital status and stigma.

**Figure 4:**
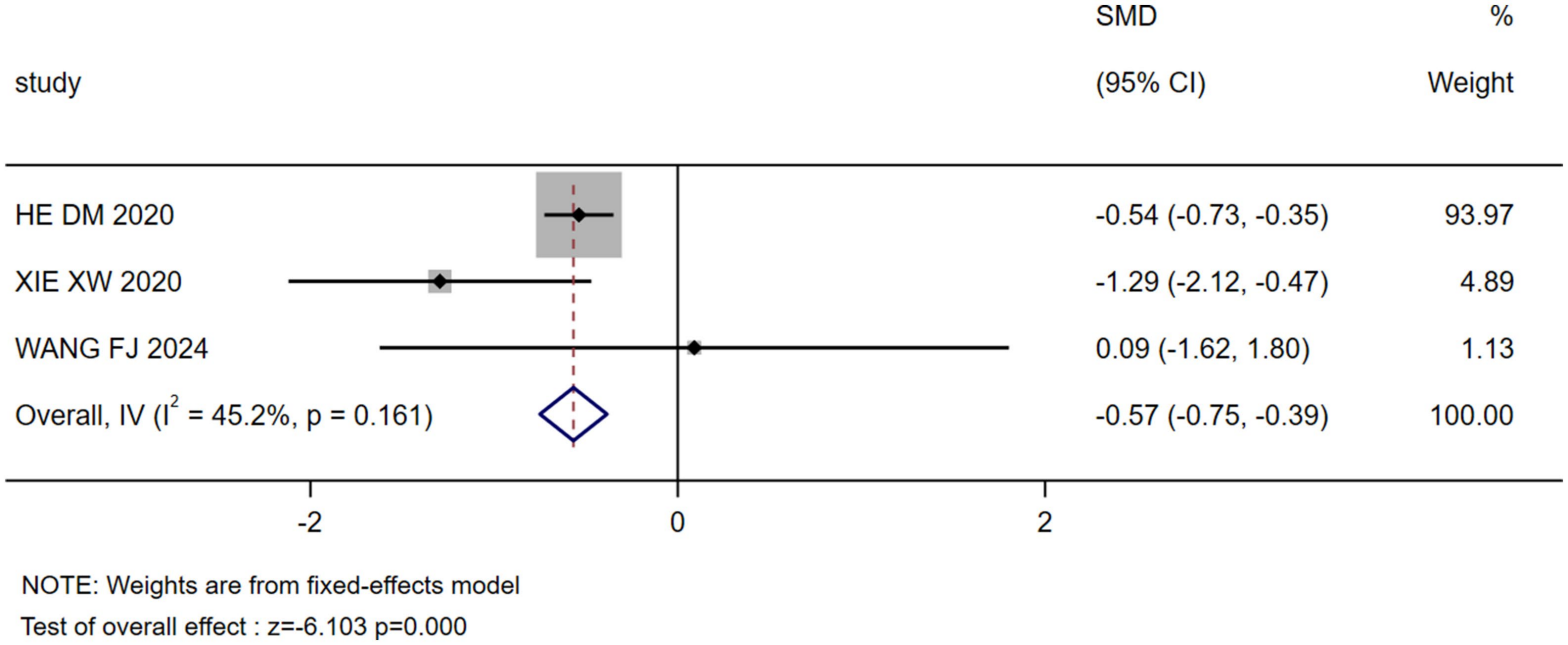
Forest plot: effect of occupation on stigma.

**Figure 5:**
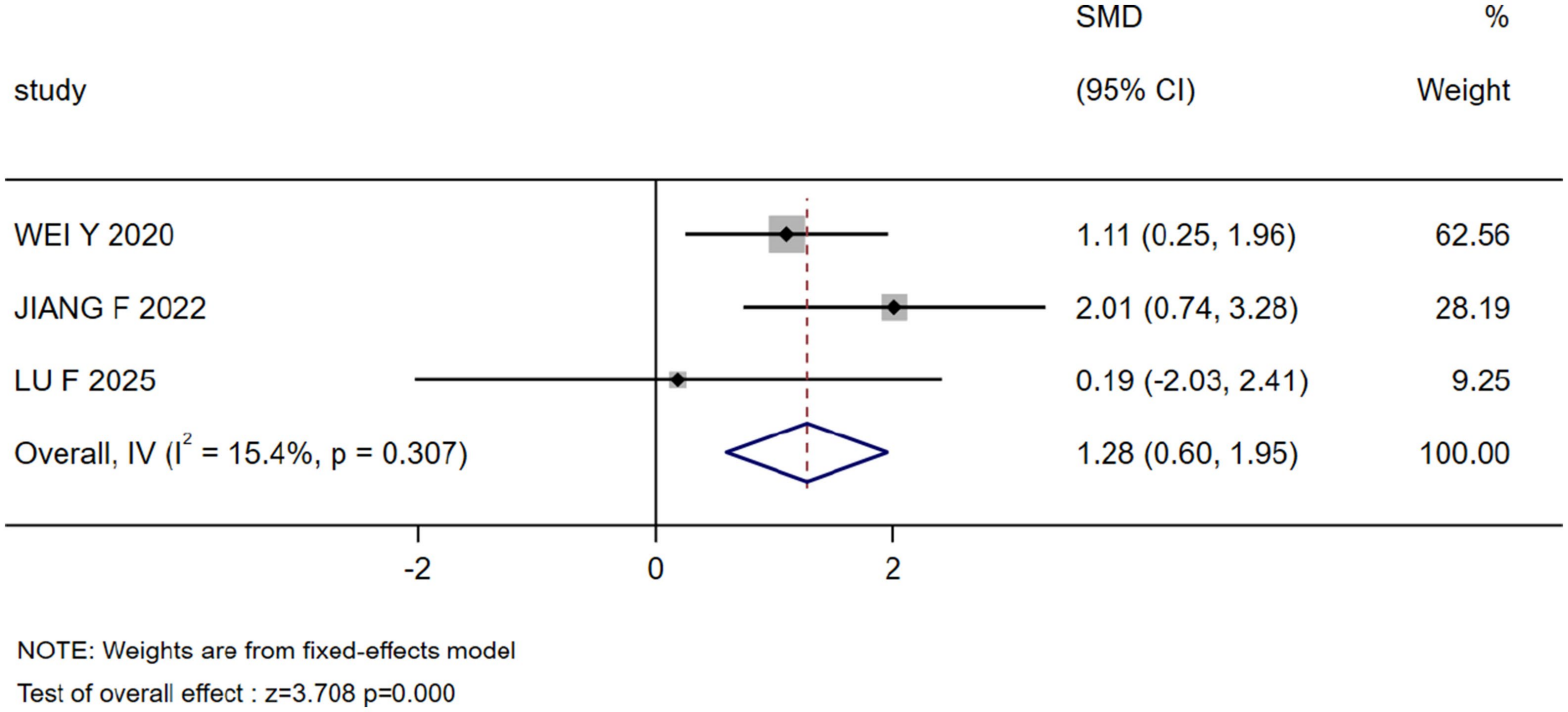
Forest plot: coping style as a predictor of stigma.

**Figure 6:**
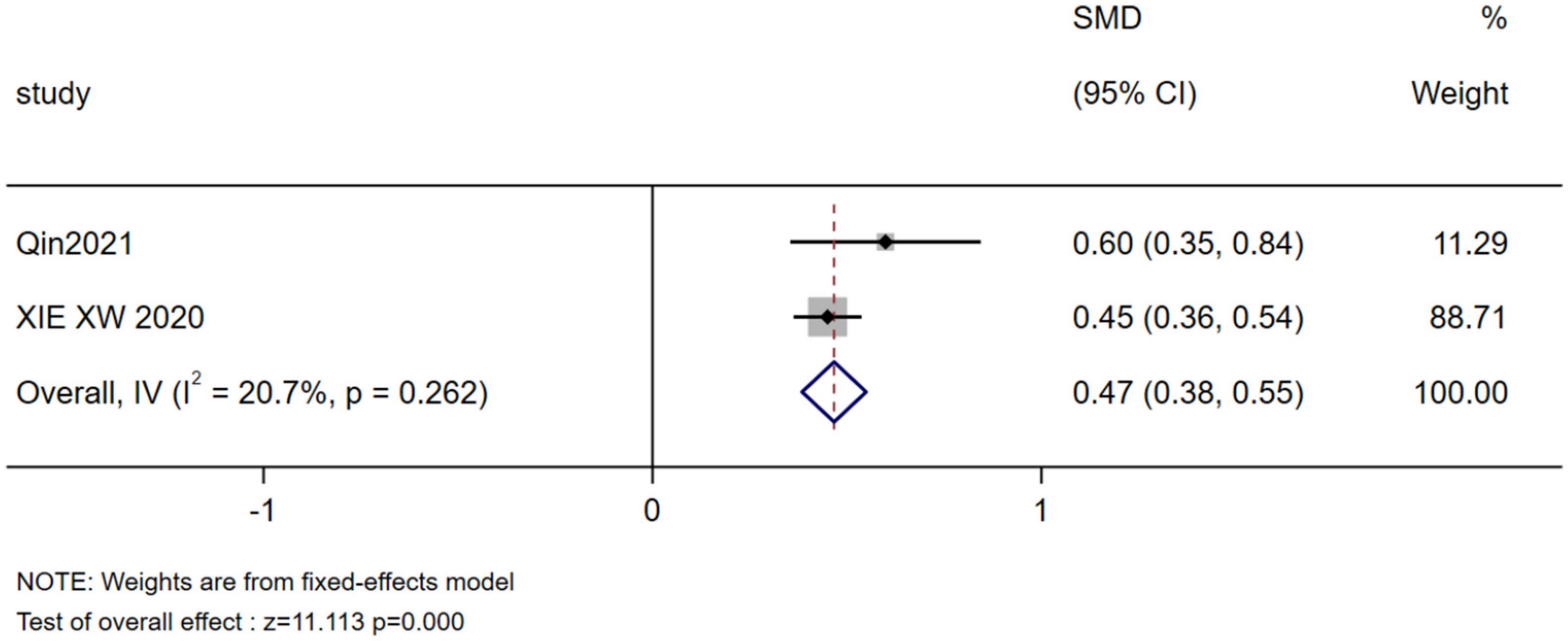
Forest plot: meta-analysis of body image impact on stigma.

**Figure 7:**
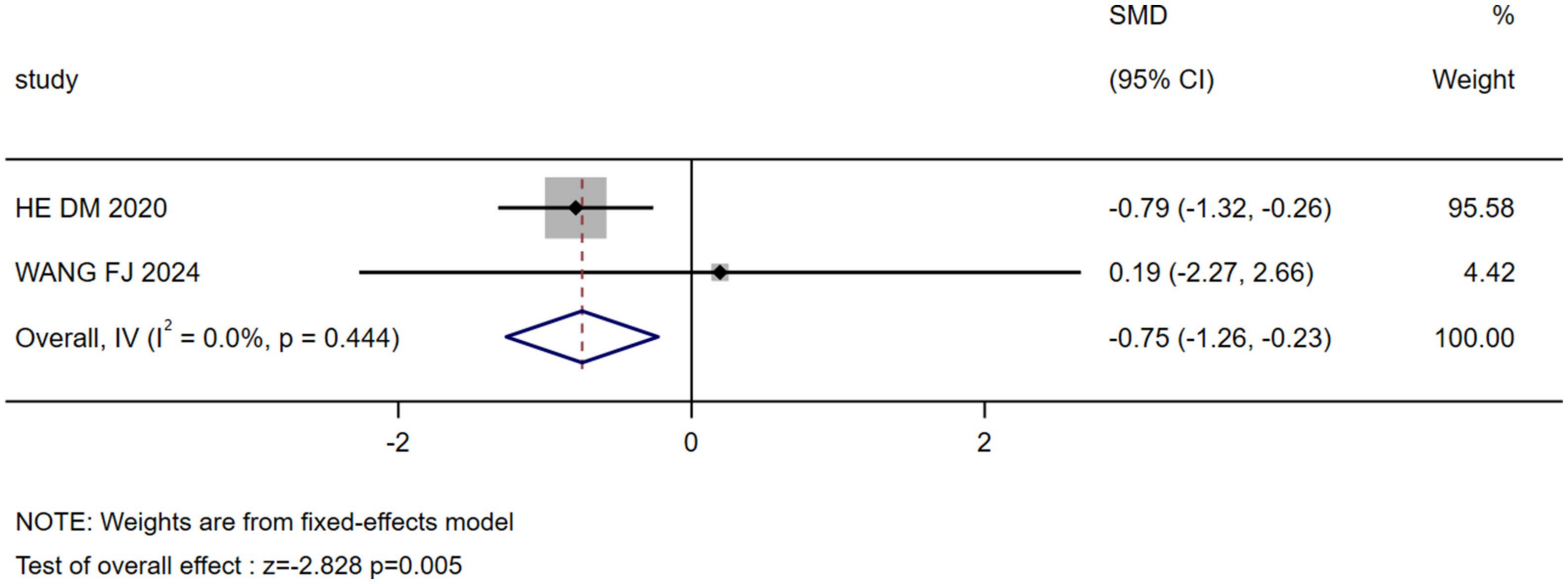
Forest plot: association of payment methods with stigma.

**Figure 8:**
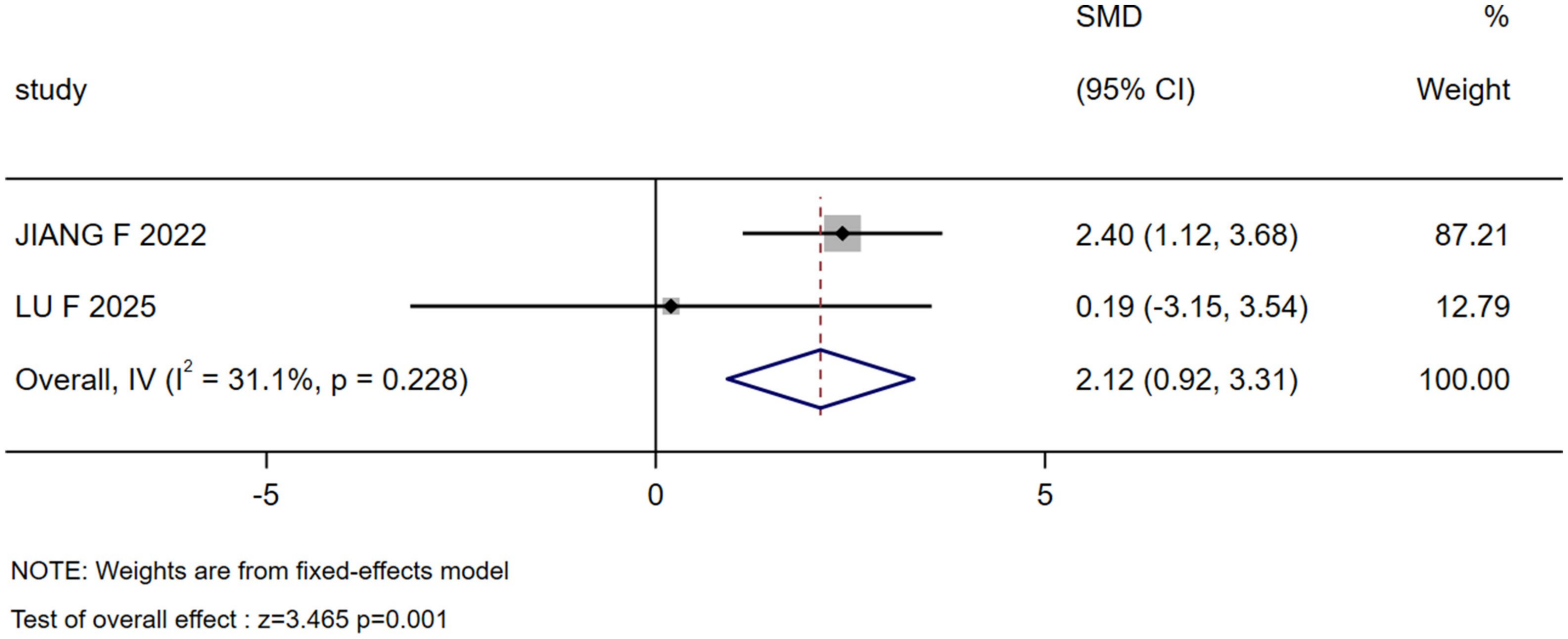
Forest plot: effect of breast reconstruction on stigma.

**Figure 9:**
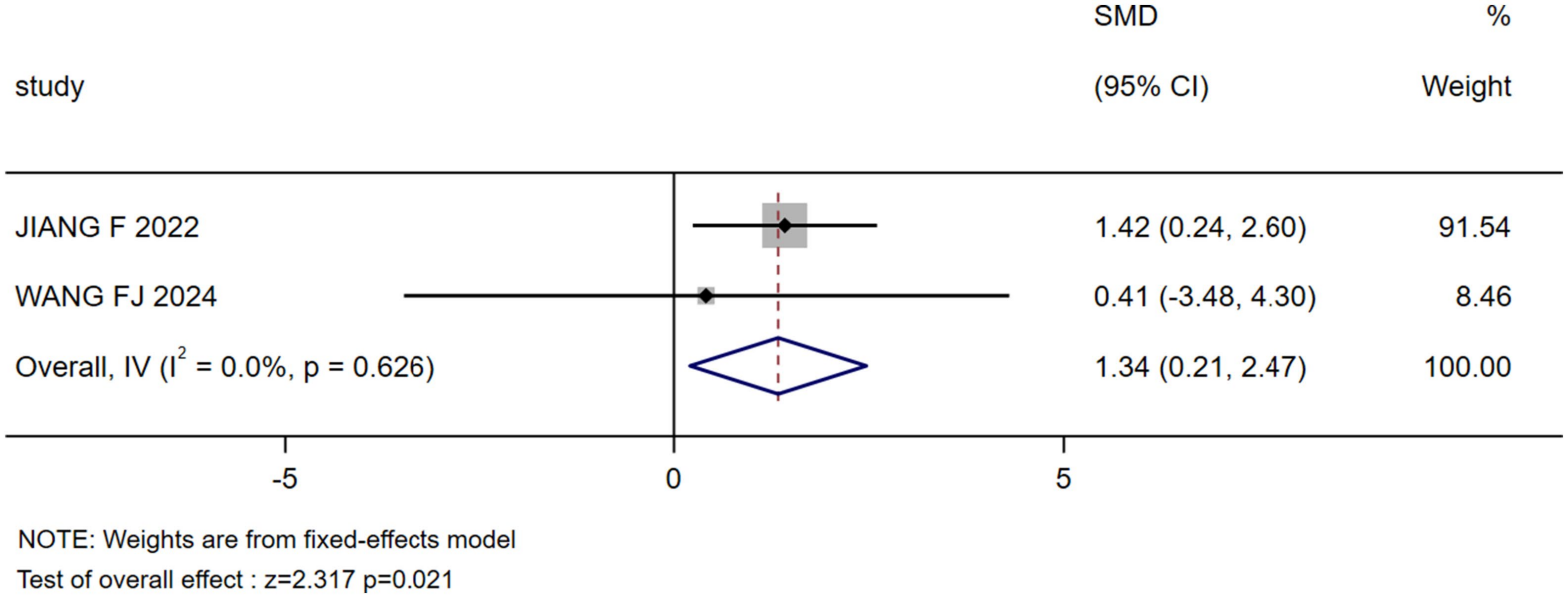
Forest plot: residential differences in stigma levels.

The results of the meta-analysis showed that marital status, occupation, coping style, body image, payment, breast reconstruction and residence were the influencing factors of stigma in postoperative breast cancer patients (*p* < 0.05), as shown in Table 3. Forest plots visualizing seven significant associations are presented in Figures 3–9.

**Table 3:** Meta-analysis of influencing factors of stigma in patients after breast cancer surgery.

Factors	Study	Heterogeneity test results		Effect model	Meta analysis results		
		*I^2^* (%)	*p*		SMD (95% CI)	*Z*	*p*
Age	8 (18, 22, 24, 25, 27, 29, 30, 33)	99.5	<0.000	Random	0.118 (−0.513, 0.750)	0.368	0.713
Education	7 (21, 24, 27–29, 32, 33)	94.9	<0.000	Random	−0.031 (−0.641, 0.580)	−0.098	0.922
Monthly income	6 (18, 21, 25, 29, 30, 32)	98.1	<0.000	Random	−0.570 (−1.589, 0.448)	−1.097	0.272
Marital status	4 (24, 28, 29, 32)	92.1	<0.000	Random	1.754(0.290, 3.218)	2.348	0.019
Postoperative time	4 (21, 25, 27, 33)	98.5	<0.000	Random	−0.739(−2.201, 0.722)	−0.991	0.322
Occupation	3 (26, 27, 30)	45.2	0.161	Fixed	−0.568(−0.750, −0.386)	−6.103	0.000
Surgery type	3 (25–27)	98.9	<0.000	Random	−2.045(−5.162, 1.073)	−1.285	0.199
Working status	3 (18, 21, 27)	95.1	<0.000	Random	0.491(−0.400, 1.382)	−1.285	0.280
Social support	3 (24, 26, 32)	97.5	<0.000	Random	0.315 (−1.181, 1.812)	0.413	0.680
Coping style	3 (22, 28, 32)	15.4	0.307	Fixed	1.276(0.601, 1.950)	3.708	0.000
Body image	2 (23, 26)	20.7	0.262	Fixed	0.467(0.384, 0.549)	11.113	0.000
Payment	2 (27, 30)	0.0	0.444	Fixed	−0.747(−1.264, −0.229)	−2.828	0.005
Breast reconstruction	2 (28, 32)	31.1	0.228	Fixed	2.116(0.919, 3.313)	3.465	0.001
Residence	2 (30, 32)	0.0	0.026	Fixed	1.337(0.206, 2.467)	2.317	0.021

##### 3.3.2.3 Sensitivity analysis

**Figure 10:**
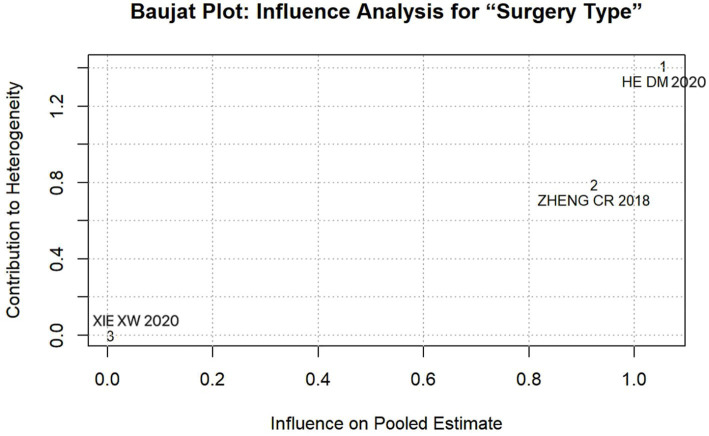
Baujat Plot: influence analysis for surgery type.

**Figure 11:**
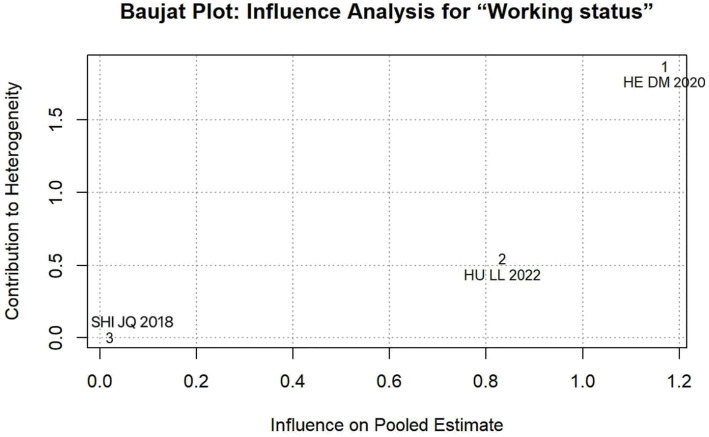
Baujat Plot: influence analysis for working status.

The results of the sensitivity analysis indicated that the effect (95% CI) and *p* value of six influencing factors, namely age, education, monthly income, surgery type, working status, and social support, underwent significant changes between fixed- and random-effects models, indicating instability (see Table 4). To pinpoint the source of this instability, we performed leave-one-out analyses (see Table 5). This procedure revealed that the study by He et al. (27) was solely responsible for the instability in the factors “surgery type” and “working status.” Specifically, the exclusion of He et al. (27) transformed the pooled effect for both factors from non-significant to statistically significant (*p* < 0.05), while simultaneously reducing heterogeneity substantially. This finding was objectively confirmed using Baujat plots for influence diagnostics (Figures 10, 11). The plots visually identified He et al. (27) as a clear outlier, located in the upper-right quadrant, indicating its strong and disproportionate influence on both the overall result and heterogeneity for these two factors. Therefore, the results for “surgery type” and “working status” should be interpreted with caution due to their dependence on a single, influential study, highlighting the need for future research to verify these associations.

**Table 4:** Sensitivity analysis of influencing factors of stigma in breast cancer patients after surgery.

Factors	Fixed effect model		Random effect model		Stability
	SMD (95% CI)	*p*	SMD (95% CI)	*p*	
Age	0.587 (0.574, 0.601)	0.000	0.118 (−0.513, 0.750)	0.713	Unstable
Education	0.526 (0.510, 0.541)	0.000	−0.031 (−0.641, 0.580)	0.922	Unstable
Monthly income	0.517 (0.501, 0.532)	0.000	−0.570 (−1.589, 0.448)	0.272	Unstable
Marital status	0.731 (0.719, 0.742)	0.000	1.754 (0.290, 3.218)	0.019	Stable
Postoperative time	0.061 (−0.045, 0.167)	0.259	−0.739 (−2.201, 0.722)	0.322	Stable
Occupation	−0.568 (−0.750, −0.386)	0.000	−0.689 (−1.258, −0.121)	0.017	Stable
Surgery type	−0.576 (−0.828, −0.324)	0.000	−2.045 (−5.162, 1.073)	0.199	Unstable
Working status	0.737 (0.548, 0.925)	0.000	0.491 (−0.400, 1.382)	0.280	Unstable
Social support	−0.251 (−0.299, −0.204)	0.000	0.315 (−1.181, 1.812)	0.680	Unstable
Coping style	1.276 (0.601, 1.950)	0.000	1.282 (0.507, 2.056)	0.001	Stable
Body image	0.467 (0.384, 0.549)	0.000	0.479 (0.364, 0.594)	0.000	Stable
Payment	−0.747 (−1.264, −0.229)	0.005	−0.747 (−1.264, −0.229)	0.005	Stable
Breast reconstruction	2.116 (0.919, 3.313)	0.001	1.862 (0.008, 3.715)	0.049	Stable
Residence	1.337 (0.206, 2.467)	0.021	1.337 (0.206, 2.467)	0.021	Stable

**Table 5:** Exclusion analysis of influencing factors of stigma instability in patients after breast cancer surgery.

Factors	Exclusion study	Before exclusion				After exclusion				Result change
		*I^2^*	Effect model	SMD (95% CI)	*p*	I*^2^*	Effect model	SMD (95% CI)	*p*	
Age	ZHENG CR (25)	99.5	Random	0.118 (−0.513, 0.750)	0.713	94.0	Random	0.249 (−0.414, 0.912)	0.461	No
Education	HE DM (27)	94.9	Random	−0.031 (−0.641, 0.580)	0.922	93.8	Random	0.085 (−0.581, 0.752)	0.802	No
Monthly income	ZHENG CR (25)	98.1	Random	−0.570 (−1.589, 0.448)	0.272	91.9	Random	0.527 (−0.002, 1.056)	0.051	No
Surgery type	HE DM (27)	98.9	Random	−2.045 (−5.162, 1.073)	0.199	93.0	Random	−3.270 (−5.297, −1.243)	0.002	Yes
Working status	HE DM (27)	95.1	Random	0.491 (−0.400, 1.382)	0.280	91.0	Random	0.963 (0.280, 1.646)	0.006	Yes
Social support	AO YT (24)	97.5	Random	0.315 (−1.181, 1.812)	0.680	60.7	Random	−0.437 (−0.935, 0.062)	0.086	No

##### 3.3.2.4 Publication bias

There were no influencing factors of ≥10 in the included literature in this study. Considering that the effectiveness of the publication bias analysis was low and had little significance when the number of included literature was less than 10, this study did not conduct publication bias analysis one by one for the influencing factors.

## 4 Discussion

### 4.1 Interpretation of results and comparison with previous research

This systematic review and meta-analysis synthesizes evidence on stigma among Chinese postoperative breast cancer patients, revealing a clinically significant burden. Pooled results from studies utilizing the Social Impact Scale (SIS) indicate a stigma score of 58.22 (95%*CI*: 55.30–61.15), which is significantly higher than that observed in Indian postoperative breast cancer patients [41.00 (*IQR*: 38.00–44.20)] (34). This finding echoes China’s distinctive “burden of silence” phenomenon—specifically, the fact that 76.7% of patients conceal their condition due to fear of discrimination (15) further underscores the profound impact of stigma within this population. Notably, this study has additionally identified seven culturally embedded influencing factors, which provide a critical perspective for explaining the uniqueness of stigma among Chinese breast cancer patients.

However, it is crucial to acknowledge that the measurement tools used to assess stigma exhibit certain limitations across the included studies. While instruments such as the SIS and Link Stigma Scale were widely used, they are general stigma measures and may not fully capture the breast cancer-specific and culturally nuanced aspects of stigma experienced by Chinese women. In China, validated disease-specific instruments such as the Breast Cancer Stigma Scale (BCSS-China) have been developed (35) and demonstrate higher sensitivity to the lived experiences of breast cancer patients. Similarly, international tools like the Breast Cancer Stigma Assessment Scale (BCSAS) have been recently validated (36). The absence of such tailored instruments in current observational studies represents a significant limitation, potentially leading to underestimation of stigma’s cultural and gender-specific dimensions. Future research should prioritize the adoption of these validated, context-sensitive tools to enhance measurement accuracy and intervention relevance.

The most striking finding concerns marital status. Contrary to some studies framing married status as a risk factor (37), unmarried/divorced/widowed Chinese postoperative breast cancer patients had significantly higher stigma than their married counterparts (*SMD* = 1.754, *p* = 0.019) in this study. We hypothesize that this inversion may stem from Confucian familial expectations, where being unmarried or losing a spouse can lead to a profound loss of social identity and support within a family-centric culture (38). Furthermore, the paradoxical finding that spousal concern intensified stigma (*β* = 1.336, *p* < 0.01) (26) could be interpreted as such support translating into implicit pressure to fulfill traditional gender roles post-mastectomy, rather than providing unconditional emotional solace. This suggests a culturally specific mechanism that warrants further qualitative investigation. Similarly, occupational engagement reduced stigma (*SMD* = −0.568, *p* < 0.001), underscoring work’s role in preserving identity—a phenomenon amplified in China’s collectivist culture where social contribution defines individual worth (39). Beyond these social and cultural correlates, body image (*SMD* = 0.467, *p* < 0.001) and breast reconstruction (*SMD* = 2.116, *p* = 0.001) emerged as core drivers of stigma. The 30% late-diagnosis rate (3, 4) necessitates radical surgeries, exacerbating “body integrity stigma.” While reconstruction alleviated this burden, its limited accessibility (especially in rural areas) reflects disparities in healthcare resource allocation. Residence-based disparities were quantifiable: rural patients suffered higher stigma (*SMD* = 1.337, *p* = 0.021), likely compounded by fragmented mental health services and entrenched cancer taboos (40).

Two findings warrant methodological reflection: First, social support showed no significant association with postoperative breast cancer stigma (*p* = 0.680), contradicting findings from Kang et al. (41), who reported that social support, as a critical coping resource, was significantly associated with reduced psychosocial distress related to cancer stigma among Korean breast cancer survivors (*r* = −0.49, *p* < 0.001). We cautiously put forward the explanation that the concept of “obligatory support” prevalent in Chinese families, where the concept of support is often intertwined with familial duties and expectations for rapid recovery, might potentially dilute its protective effect. This hypothesis regarding the qualitative nature, rather than the quantity, of support could explain the discrepancy and should be tested in future research. Second, economic factors showed differential effects: while monthly income level was not significantly associated with stigma (*SMD* = −0.570, *p* = 0.272), self-payment status emerged as a significant predictor (*SMD* = −0.747, *p* = 0.005). This suggests that economic influence may operate indirectly financial toxicity from out-of-pocket expenses could amplify perceived familial burden, particularly within the context of Chinese familial responsibility ethics (42).

It is also worth noting that the conceptual understanding of stigma in breast cancer has been advanced by recent theoretical work. Wu et al. (43) conducted a concept analysis specific to breast cancer patients, identifying key attributes such as impaired body image, negative stereotypes, avoidance, and experienced discrimination. Their work provides a valuable theoretical framework that aligns with several of our empirical findings, particularly regarding the roles of body image and social isolation. Incorporating this conceptual model helps contextualize our results within a broader theoretical narrative and strengthens the interpretative depth of this review. Furthermore, while our study focuses specifically on postoperative patients, it is important to acknowledge that the vast majority of women diagnosed with localized breast cancer undergo surgery as part of standard treatment (10). This contextual detail reinforces the relevance and generalizability of our findings to the broader population of breast cancer patients in China. Finally, a recent meta-analysis examined correlates of stigma across a broader range of cancer types (44). While that review included breast cancer patients, it did not focus specifically on the postoperative phase nor on the Chinese cultural context. Our study thus adds value by providing a more targeted analysis of the unique factors operating in this specific subgroup and setting.

### 4.2 Implications for practice, policy and research

For clinical practice, culturally adapted interventions are imperative: Psychological support should prioritize unmarried/divorced women through acceptance-commitment therapy to address identity crises, while integrating spouses into counseling to reframe care as empowerment rather than obligation (45). Concurrently, surgical decision-making must emphasize shared deliberation on breast reconstruction, highlighting its psychosocial benefits, with standardized preoperative protocols to address body image concerns (46).

Policy reforms must bridge structural gaps: Expanding insurance reimbursement to cover reconstruction surgery and evidence-based psychosocial interventions (e.g., cognitive-behavioral therapy) is critical. Pilot programs integrating mental health services into rural cancer survivorship care should be prioritized. Anti-stigma efforts require media collaboration to normalize breast cancer narratives, potentially adopting strategies from established campaigns like Hong Kong’s Pink Revolution (47).

Research priorities should center on mechanistic studies that employ mixed methods to unpack paradoxes, intervention trials that develop stepped-care models, and longitudinal designs that track stigma trajectories from the point of diagnosis through to survivorship.

### 4.3 Strengths and limitations

This study demonstrates significant strengths. Methodologically, it strictly adhered to the PRISMA statement and AHRQ criteria, with all included studies rated moderate-to-high quality (Grades A/B). The dual-reviewer screening and data extraction process, augmented by third-party arbitration for discrepancies, enhanced reliability. Substantively, it represents the first systematic review and meta-analysis focusing exclusively on postoperative breast cancer patients in mainland China, addressing the critical gap of cultural specificity absent in international models. Furthermore, robust sensitivity analyses confirmed the stability of seven core influencing factors (marital status, occupation, coping style, body image, payment method, breast reconstruction, residence), reinforcing their credibility.

However, limitations merit consideration: (1) Causality and Design Limitation: The cross-sectional design of all included studies prevents definitive causal conclusions regarding the relationships between influencing factors and stigma. Furthermore, the variability in the definition of the “postoperative” phase across studies may have introduced heterogeneity, as stigma mechanisms may differ across these phases. (2) Heterogeneity and Measurement Limitations: Significant unresolved heterogeneity was observed for certain factors such as surgery type and working status. While sensitivity analyses improved stability after excluding outlier studies, these associations require further validation. Additionally, the reliance on general stigma scales (e.g., SIS, Link) rather than breast cancer-specific instruments (e.g., BCSS-China, BCSAS) may have limited the cultural and clinical sensitivity of stigma measurement. (3) Publication Bias Assessment Limitation: In accordance with Cochrane guidelines, this study only assessed potential publication bias via funnel plot asymmetry for factors where ≥10 studies were available. For the majority of influencing factors with fewer studies, statistical evaluation of small-study effects was not feasible, and thus the results for these factors should be interpreted with appropriate caution.

## 5 Conclusion

This systematic review and meta-analysis confirms that Chinese postoperative breast cancer patients face a significant stigma burden, which is higher than in some other populations and linked to China’s “burden of silence” due to fear of discrimination. Key culturally specific influencing factors include marital status, occupational engagement, coping style, body image, breast reconstruction, rural residence, and self-payment status, with paradoxical effects noted in spouses’ attitudes and social support. These findings highlight the need for culturally adapted clinical interventions, policy reforms, and further research to mitigate stigma and improve patients’ psychosocial well-being.

## Data Availability

The original contributions presented in the study are included in the article/Supplementary material, further inquiries can be directed to the corresponding authors.

## References

[1] Bray F Laversanne M Sung H Ferlay J Siegel RL Soerjomataram I . Global cancer statistics 2022: GLOBOCAN estimates of incidence and mortality worldwide for 36 cancers in 185 countries. CA Cancer J Clin. (2024) 74:229–63. doi: 10.3322/caac.21834, PMID: 38572751

[2] Han B Zheng R Zeng H Wang S Sun K Chen R . Cancer incidence and mortality in China, 2022. J Natl Cancer Cent. (2024) 4:47–53. doi: 10.1016/j.jncc.2024.01.006, PMID: 39036382 PMC11256708

[3] Zhang X Yang L Liu S Cao LL Wang N Li HC . Interpretation on the report of global cancer statistics 2022. Zhonghua Zhong Liu Za Zhi. (2024) 46:710. doi: 10.3760/cma.j.cn112152-20240416-00152 39034807

[4] Arnold M Morgan E Rumgay H Mafra A Singh D Laversanne M . Current and future burden of breast cancer: global statistics for 2020 and 2040. Breast. (2022) 66:15–23. doi: 10.1016/j.breast.2022.08.010. Epub 2022 Sep 2, PMID: 36084384 PMC9465273

[5] Cao MD. Interpretation on the global cancer statistics of GLOBOCAN 2022. Chin J Front Med Sci. (2024) 16:1–5. doi: 10.12037/YXQY.2024.06-01

[6] Zheng RS Chen R Han BF Wang SM Li L Sun KX . Cancer incidence and mortality in China, 2022. Zhonghua Zhong Liu Za Zhi. (2024) 46:221–31. doi: 10.3760/cma.j.cn112152-20240119-00035 38468501

[7] Gradishar WJ Moran MS Abraham J Abramson V Aft R Agnese D . Breast cancer, version 3.2024, NCCN clinical practice guidelines in oncology. J Natl Compr Cancer Netw. (2024) 22:331–57. doi: 10.6004/jnccn.2024.0035, PMID: 39019058

[8] Tao X Li T Gandomkar Z Brennan PC Reed WM. Incidence, mortality, survival, and disease burden of breast cancer in China compared to other developed countries. Asia Pac J Clin Oncol. (2023) 19:645–54. doi: 10.1111/ajco.13958, PMID: 37026375

[9] Hou LJ Liu LY Wang F Yu LX Yu ZG. Psychological problems in breast cancer patients should be taken seriously. Zhonghua Wai Ke Za Zhi. (2024) 62:110–5. doi: 10.3760/cma.j.cn112139-20231016-00175, PMID: 38310377

[10] Miller KD Nogueira L Devasia T Mariotto AB Yabroff KR Jemal A . Cancer treatment and survivorship statistics, (2022). CA Cancer J Clin. (2022) 72:409–36. doi: 10.3322/caac.21731, PMID: 35736631

[11] Greenlee H DuPont-Reyes MJ Balneaves LG Carlson LE Cohen MR Deng G . Clinical practice guidelines on the evidence-based use of integrative therapies during and after breast cancer treatment. CA Cancer J Clin. (2017) 67:194–232. doi: 10.3322/caac.21397, PMID: 28436999 PMC5892208

[12] Khan TM Leong JP Ming LC Khan AH. Association of knowledge and cultural perceptions of Malaysian women with delay in diagnosis and treatment of breast cancer: a systematic review. Asian Pac J Cancer Prev. (2015) 16:5349–57. doi: 10.7314/apjcp.2015.16.13.5349, PMID: 26225677

[13] Fan ZM. (2023). Case work intervention study on reducing stigma in cancer patients. Wuhan, China: Huazhong Agricultural University.

[14] Amini-Tehrani M Zamanian H Daryaafzoon M Andikolaei S Mohebbi M Imani A . Body image, internalized stigma and enacted stigma predict psychological distress in women with breast cancer: a serial mediation model. J Adv Nurs. (2021) 77:3412–23. doi: 10.1111/jan.14881 33969915

[15] Fujisawa D Umezawa S Fujimori M Miyashita M. Prevalence and associated factors of perceived cancer-related stigma in Japanese cancer survivors. Jpn J Clin Oncol. (2020) 50:1325–9. doi: 10.1093/jjco/hyaa135, PMID: 32776092

[16] Kong RH. (2015). The status and influencing factors of stigma in young patients with breast cancer. Jinan, China: Shandong University.

[17] Xiao HM Zhou XY Zhang XJ Li N Li XZ. Influencing factors of stigma in breast cancer patients after operation and its relationship with self-esteem, quality of life and psychosocial adaptability. Prog in Mod Biomed. (2021) 21:4522–6. doi: 10.13241/j.cnki.pmb.2021.23.026

[18] Shi JQ Ma JL Hu FH Ye HZ. Correlation of stigma, coping style and self-esteem among patients after breast modified radical mastectomy. Chin J Mod Nurs. (2018) 24:332–5. doi: 10.3760/cma.j.issn.1674-2907.2018.03.020

[19] Page MJ McKenzie JE Bossuyt PM Boutron I Hoffmann TC Mulrow CD . The PRISMA 2020 statement: an updated guideline for reporting systematic reviews. BMJ. (2021) 29:372. doi: 10.1136/bmj.n71 PMC8005924 33782057

[20] Dougherty DM Agency for Healthcare Research and Quality. Alphascript Publishing (2001) 73:81–83.

[21] Hu LL Zhou XF Wang YB Zhou Y Ren HH Yao F. Influencing factors of stigma after modified radical mastectomy in patients with breast cancer. Chin Med Her (2022) 19:100–103. Chinese.

[22] Wei Y Chen LL Tao FL. Correlation between stigma, coping style and self-esteem in patients undergoing modified radical mastectomy for breast cancer. Nurs Prac Res. (2020) 17:78–80. doi: 10.3969/j.issn.1672-9676.2020.04.029

[23] Qin ZW Shen C Wei WF Chen SQ. Relationship between self-identity, self-image and stigma of breast cancer patients. Nurs Rehabil Mag. (2021) 20:68–71. doi: 10.3969/j.issn.1671-9875.2021.11.019

[24] Ao YT. Analysis of the current situation and risk factors of postoperative stigma in patients with breast cancer surgery. Med Equip (2023) 36:145–147. Chinese. doi: 10.3969/j.issn.1002-2376.2023.01.047

[25] Zheng CR Wang HZ. Analysis of stigma status and influencing factors of breast cancer patients after operation. J Nurs (2018) 25:7–9. Chinese. doi: 10.16460/j.issn1008-9969.2018.02.007

[26] Xie XW. (2020). Study on the relationship between body image level, social support and stigma in patients with breast cancer after surgery. Jilin, China: Yanbian University.

[27] He DM. Study on the mediating effect of coping modes between patients’ stigma and marital quality after radical mastectomy. Nanchong: Chuanbei Medical College (2020).

[28] Lu F Jiao SS Lu SC Niu ZZ. Analysis of related factors of stigma in patients with early breastcancer after radical mastectomy based on linear regressionand to formulate intervention measures. J Med Forum. (2025) 46:1034–8. doi: 10.20159/j.cnki.jmf.2025.10.006

[29] Peng XY. Investigation on the status Ouo of stigma and its analysis of influencing factors in patients with BreastCancer after modified radical mastectomy. Refl Rehabil Med. (2025) 6:171–3. doi: 10.16344/j.cnki.10-1669/r4.2025.02.044.29

[30] Wang FJ. A study on postoperative stigma, self-efficacy and quality of life in patients with breast. Gansu: Gansu University of Chinese Medicine (2024).

[31] Wang JH Bai P Wei YT. The status of postoperative stigma of breast cancer patients and its correlation with self-compassion in a grade-a tertiary hospital in Beijing. Med Soc. (2024) 37:138–44. doi: 10.13723/j.yxysh.2024.06.020

[32] Jiang F Zhou F Xiao H. The related factors of disgust feeling after radical correction breast cancer in the young and mid aged patients. J Int Psychl. (2022) 49:1074–7. doi: 10.13479/j.cnki.jip.2022.06.001

[33] Li X.. (2023). The study on the status quo and influencing factors of shame sense in postoperative patients with breast cancer and its correlation with negative emotions and coping styles. Jilin, China: Beihua University.

[34] Tripathi L Datta SS Agrawal SK Chatterjee S Ahmed R. Stigma perceived by women following surgery for breast Cancer. Indian J Med Paediatr Oncol. (2017) 38:146–52. doi: 10.4103/ijmpo.ijmpo_74_16, PMID: 28900322 PMC5582551

[35] Bu X Li S Cheng ASK Ng PHF Xu X Xia Y . Breast Cancer stigma scale: a reliable and valid stigma measure for patients with breast Cancer. Front Psychol. (2022) 13:841280. doi: 10.3389/fpsyg.2022.841280, PMID: 35756211 PMC9226439

[36] Cenit-García J Buendia-Gilabert C Contreras-Molina C Puente-Fernández D Fernández-Castillo R García-Caro MP. Development and psychometric validation of the breast Cancer stigma assessment scale for women with breast Cancer and its survivors. Healthcare (Basel). (2024) 12:420. doi: 10.3390/healthcare12040420, PMID: 38391796 PMC10887980

[37] Yıldız K Koç Z. Stigmatization, discrimination and illness perception among oncology patients: a cross-sectional and correlational study. Eur J Oncol Nurs. (2021) 54:102000. doi: 10.1016/j.ejon.2021.102000, PMID: 34492525

[38] Jiang C Wang Y Jiang W Cai J Wang L Wu X. Metaphors and stigma in Confucian culture: a qualitative study of Cancer risk communication dilemmas for Cascade screening among hereditary Cancer families from China. Psychooncology. (2025) 34:e70205. doi: 10.1002/pon.70205, PMID: 40509552 PMC12163203

[39] Mao B Shen Y Chen Y Zhou P Pan Y. Experiences of healthcare professionals returning to work post breast cancer diagnosis in China: a descriptive qualitative study. Sci Rep. (2025) 15:1938. doi: 10.1038/s41598-024-82893-8, PMID: 39809839 PMC11733254

[40] Jiang MF Gao J Yang L Lu YM Qian J Li JZ. The mediating role of loneliness and social support between stigma and social avoidance in rural breast cancer survivors. Chin J Gen Pract. (2023) 21:2000–4. doi: 10.16766/j.cnki.issn.1674-4152.003276

[41] Kang NE Kim HY Kim JY Kim SR. Relationship between cancer stigma, social support, coping strategies and psychosocial adjustment among breast cancer survivors. J Clin Nurs. (2020) 29:4368–78. doi: 10.1111/jocn.15475, PMID: 32860289

[42] Hu RY Wang JY Chen WL Zhao J Shao CH Wang JW . Stress, coping strategies and expectations among breast cancer survivors in China: a qualitative study. BMC Psychol. (2021) 9:26. doi: 10.1186/s40359-021-00515-8, PMID: 33557956 PMC7869238

[43] Wu J Zeng N Wang L Yao L. The stigma in patients with breast cancer: a concept analysis. Asia Pac J Oncol Nurs. (2023) 10:100293. doi: 10.1016/j.apjon.2023.100293, PMID: 37886719 PMC10597826

[44] Tang WZ Yusuf A Jia K Iskandar YHP Mangantig E Mo XS . Correlates of stigma for patients with breast cancer: a systematic review and meta-analysis. Support Care Cancer. (2022) 31:55. doi: 10.1007/s00520-022-07506-4, PMID: 36526859

[45] Mesko B deBronkart D Dhunnoo P Arvai N Katonai G Riggare S. The evolution of patient empowerment and its impact on health care's future. J Med Internet Res. (2025) 27:e60562. doi: 10.2196/60562, PMID: 40311140 PMC12082052

[46] Williams T Fine K Duckworth E Adam T Bozigar C McFarland A . Patient decision aids in breast surgery and breast reconstruction reduce decisional conflict: a systematic review and meta-analysis. Breast Cancer Res Treat. (2025) 213:1–14. doi: 10.1007/s10549-025-07752-0, PMID: 40588633 PMC12259761

[47] Hong Kong Cancer Fund (2020) Shop for charity in pink revolution’s “shop for pink” programme. Hong Kong: Hong Kong Cancer Fund. Available online at: https://www.cancer-fund.org/en/blog/shop-charity-pink-revolutions-shop-pink-programme/

