# Supplementary material for: Factors influencing stigma in Chinese postoperative breast cancer patients: a systematic review and meta-analysis
Source: Front Med (Lausanne). 2025 Oct 9;12:1681487. doi: 10.3389/fmed.2025.1681487 (PMC12546193; doi:10.3389/fmed.2025.1681487)
Supplement: Supplementary file 1 [file Table_1.docx]

**Supplementary Material 1**——Search formula and related information

| SinoMed |  |
| --- | --- |
| ("乳腺癌"[全部字段:智能] OR "乳岩"[全部字段:智能] OR "乳腺肿瘤"[全部字段:智能] OR "乳癌"[全部字段:智能]) AND ("病耻感"[全部字段:智能] OR "内在病耻感"[全部字段:智能] OR "外在病耻感"[全部字段:智能] OR "自我耻辱感"[全部字段:智能] OR "羞耻感"[全部字段:智能]) AND [("影响因素"[全部字段:智能] OR "相关因素"[全部字段:智能] OR "危险因素"[全部字段:智能] OR "预测因素"[全部字段:智能] OR "现状"[全部字段:智能] OR "调查"[全部字段:智能] OR "相关性"[全部字段:智能])](javascript:toDoRelimitSearch();) | 65 |
| CNKI |  |
| SU=(乳腺癌+乳岩+乳腺肿瘤+乳癌) AND SU=(病耻感+内在病耻感+外在病耻感+自我耻辱感+羞耻感) AND SU=(影响因素+相关因素+危险因素+预测因素+现状+调查+相关性) “同义词扩展” | 74 |
| WanFang Data |  |
| 主题:(乳腺癌 or 乳岩 or 乳腺肿瘤 or 乳癌) and 主题:(病耻感 or 内在病耻感 or 外在病耻感 or 自我耻辱感 or 羞耻感) and 主题:(影响因素 or 相关因素 or 危险因素 or 预测因素 or 现状 or 调查 or 相关性) “同义词扩展” | 103 |
| VIP |  |
| U=(乳腺癌 OR 乳岩 OR 乳腺肿瘤 OR 乳癌) AND U=(病耻感 OR 内在病耻感 OR 外在病耻感 OR 自我耻辱感 OR 羞耻感) AND U=(影响因素 OR 相关因素 OR 危险因素 OR 预测因素 OR 现状 OR 调查 OR 相关性) | 107 |
| The Cochrane Library |  |
| ((Breast Neoplasm) OR (Neoplasm, Breast) OR (Breast Tumors) OR (Breast Tumor) OR (Tumor, Breast) OR (Tumors, Breast) OR (Neoplasms, Breast) OR (Breast Cancer) OR (Cancer, Breast) OR (Mammary Cancer) OR (Cancer, Mammary) OR (Cancers, Mammary) OR (Mammary Cancers) OR (Malignant Neoplasm of Breast) OR (Breast Malignant Neoplasm) OR (Breast Malignant Neoplasms) OR (Malignant Tumor of Breast) OR (Breast Malignant Tumor) OR (Breast Malignant Tumors) OR (Cancer of Breast) OR (Cancer of the Breast) OR (Mammary Carcinoma, Human) OR (Carcinoma, Human Mammary) OR (Carcinomas, Human Mammary) OR (Human Mammary Carcinomas) OR (Mammary Carcinomas, Human) OR (Human Mammary Carcinoma) OR (Mammary Neoplasms, Human) OR (Human Mammary Neoplasm) OR (Human Mammary Neoplasms) OR (Neoplasm, Human Mammary) OR (Neoplasms, Human Mammary) OR (Mammary Neoplasm, Human) OR (Breast Carcinoma) OR (Breast Carcinomas) OR (Carcinoma, Breast) OR (Carcinomas, Breast)):ti,ab,kw AND ((Social Stigma) OR (Social Stigmas) OR (Stigmas, Social) OR (Stigma, Social) OR (Stigma) OR (internalized stigma) OR (externalized stigma) OR (selfstigma) OR (shame)):ti,ab,kw AND ((influence factors) OR (related factors) OR (risk factors) OR (predictive factors) OR (current situation) OR (investigation) OR (relevance)):ti,ab,kw | 30 |
| PubMed |  |
| (((((((((((((((((((((((((((((((((((((((Breast Neoplasm[Title/Abstract]) OR (Neoplasm, Breast[Title/Abstract])) OR (Breast Tumors[Title/Abstract])) OR (Breast Tumor[Title/Abstract])) OR (Tumor, Breast[Title/Abstract])) OR (Tumors, Breast[Title/Abstract])) OR (Neoplasms, Breast[Title/Abstract])) OR (Breast Cancer[Title/Abstract])) OR (Cancer, Breast[Title/Abstract])) OR (Mammary Cancer[Title/Abstract])) OR (Cancer, Mammary[Title/Abstract])) OR (Cancers, Mammary[Title/Abstract])) OR (Mammary Cancers[Title/Abstract])) OR (Malignant Neoplasm of Breast[Title/Abstract])) OR (Breast Malignant Neoplasm[Title/Abstract])) OR (Breast Malignant Neoplasms[Title/Abstract])) OR (Malignant Tumor of Breast[Title/Abstract])) OR (Breast Malignant Tumor[Title/Abstract])) OR (Breast Malignant Tumors[Title/Abstract])) OR (Cancer of Breast[Title/Abstract])) OR (Cancer of the Breast[Title/Abstract])) OR (Mammary Carcinoma, Human[Title/Abstract])) OR (Carcinoma, Human Mammary[Title/Abstract])) OR (Carcinomas, Human Mammary[Title/Abstract])) OR (Human Mammary Carcinomas[Title/Abstract])) OR (Mammary Carcinomas, Human[Title/Abstract])) OR (Human Mammary Carcinoma[Title/Abstract])) OR (Mammary Neoplasms, Human[Title/Abstract])) OR (Human Mammary Neoplasm[Title/Abstract])) OR (Human Mammary Neoplasms[Title/Abstract])) OR (Neoplasm, Human Mammary[Title/Abstract])) OR (Neoplasms, Human Mammary[Title/Abstract])) OR (Mammary Neoplasm, Human[Title/Abstract])) OR (Breast Carcinoma[Title/Abstract])) OR (Breast Carcinomas[Title/Abstract])) OR (Carcinoma, Breast[Title/Abstract])) OR (Carcinomas, Breast[Title/Abstract])) OR (Breast Neoplasm[MeSH Terms])) AND ((Social Stigma[MeSH Terms]) OR ((((((((Social Stigmas[Title/Abstract]) OR (Stigmas, Social[Title/Abstract])) OR (Stigma, Social[Title/Abstract])) OR (Stigma[Title/Abstract])) OR (internalized stigma[Title/Abstract])) OR (externalized stigma[Title/Abstract])) OR (selfstigma[Title/Abstract])) OR (shame[Title/Abstract])))) AND (((((((influence factors[Title/Abstract]) OR (related factors[Title/Abstract])) OR (risk factors[Title/Abstract])) OR (predictive factors[Title/Abstract])) OR (current situation[Title/Abstract])) OR (investigation[Title/Abstract])) OR (relevance[Title/Abstract])) | 39 |
| EMBase |  |
| #1 'Breast Neoplasm' OR 'Neoplasm, Breast' OR 'Breast Tumors' OR 'Breast Tumor' OR 'Tumor, Breast' OR 'Tumors, Breast' OR 'Neoplasms, Breast' OR 'Breast Cancer' OR 'Cancer, Breast' OR 'Mammary Cancer' OR 'Cancer, Mammary' OR 'Cancers, Mammary' OR 'Mammary Cancers' OR 'Malignant Neoplasm of Breast' OR 'Breast Malignant Neoplasm' OR 'Breast Malignant Neoplasms' OR 'Malignant Tumor of Breast' OR 'Breast Malignant Tumor' OR 'Breast Malignant Tumors' OR 'Cancer of Breast' OR 'Cancer of the Breast' OR 'Mammary Carcinoma, Human' OR 'Carcinoma, Human Mammary' OR 'Carcinomas, Human Mammary' OR 'Human Mammary Carcinomas' OR 'Mammary Carcinomas, Human' OR 'Human Mammary Carcinoma' OR 'Mammary Neoplasms, Human' OR 'Human Mammary Neoplasm' OR 'Human Mammary Neoplasms' OR 'Neoplasm, Human Mammary' OR 'Neoplasms, Human Mammary' OR 'Mammary Neoplasm, Human' OR 'Breast Carcinoma' OR 'Breast Carcinomas' OR 'Carcinoma, Breast' OR 'Carcinomas, Breast'  #2 'Social Stigma' OR 'Social Stigmas' OR 'Stigmas, Social' OR 'Stigma, Social' OR 'Stigma' OR 'internalized stigma' OR 'externalized stigma' OR 'selfstigma' OR 'shame'  #3 'influence factors' OR 'related factors' OR 'risk factors' OR 'predictive factors' OR 'current situation' OR 'investigation' OR 'relevance'  #4 #1 AND #2 AND #3 | 87 |
| Scopus |  |
| #1 TITLE-ABS-KEY("Breast Neoplasm" OR "Neoplasm, Breast" OR "Breast Tumors" OR "Breast Tumor" OR "Tumor, Breast" OR "Tumors, Breast" OR "Neoplasms, Breast" OR "Breast Cancer" OR "Cancer, Breast" OR "Mammary Cancer" OR "Cancer, Mammary" OR "Cancers, Mammary" OR "Mammary Cancers" OR "Malignant Neoplasm of Breast" OR "Breast Malignant Neoplasm" OR "Breast Malignant Neoplasms" OR "Malignant Tumor of Breast" OR "Breast Malignant Tumor" OR "Breast Malignant Tumors" OR "Cancer of Breast" OR "Cancer of the Breast" OR "Mammary Carcinoma, Human" OR "Carcinoma, Human Mammary" OR "Carcinomas, Human Mammary" OR "Human Mammary Carcinomas" OR "Mammary Carcinomas, Human" OR "Human Mammary Carcinoma" OR "Mammary Neoplasms, Human" OR "Human Mammary Neoplasm" OR "Human Mammary Neoplasms" OR "Neoplasm, Human Mammary" OR "Neoplasms, Human Mammary" OR "Mammary Neoplasm, Human" OR "Breast Carcinoma" OR "Breast Carcinomas" OR "Carcinoma, Breast" OR "Carcinomas, Breast")  #2 TITLE-ABS-KEY("Social Stigma" OR "Social Stigmas" OR "Stigmas, Social" OR "Stigma, Social" OR "Stigma" OR "internalized stigma" OR "externalized stigma" OR "selfstigma" OR "shame")  #3 TITLE-ABS-KEY("influence factors" OR "related factors" OR "risk factors" OR "predictive factors" OR "current situation" OR "investigation" OR "relevance")  #4 #1 AND #2 AND #3 | 118 |
| Web of Science |  |
| #1 "Breast Neoplasm" OR "Neoplasm, Breast" OR "Breast Tumors" OR "Breast Tumor" OR "Tumor, Breast" OR "Tumors, Breast" OR "Neoplasms, Breast" OR "Breast Cancer" OR "Cancer, Breast" OR "Mammary Cancer" OR "Cancer, Mammary" OR "Cancers, Mammary" OR "Mammary Cancers" OR "Malignant Neoplasm of Breast" OR "Breast Malignant Neoplasm" OR "Breast Malignant Neoplasms" OR "Malignant Tumor of Breast" OR "Breast Malignant Tumor" OR "Breast Malignant Tumors" OR "Cancer of Breast" OR "Cancer of the Breast" OR "Mammary Carcinoma, Human" OR "Carcinoma, Human Mammary" OR "Carcinomas, Human Mammary" OR "Human Mammary Carcinomas" OR "Mammary Carcinomas, Human" OR "Human Mammary Carcinoma" OR "Mammary Neoplasms, Human" OR "Human Mammary Neoplasm" OR "Human Mammary Neoplasms" OR "Neoplasm, Human Mammary" OR "Neoplasms, Human Mammary" OR "Mammary Neoplasm, Human" OR "Breast Carcinoma" OR "Breast Carcinomas" OR "Carcinoma, Breast" OR "Carcinomas, Breast"  #2 "Social Stigma" OR "Social Stigmas" OR "Stigmas, Social" OR "Stigma, Social" OR "Stigma" OR "internalized stigma" OR "externalized stigma" OR "selfstigma" OR "shame"  #3 "influence factors" OR "related factors" OR "risk factors" OR "predictive factors" OR "current situation" OR "investigation" OR "relevance"  #4 #1 AND #2 AND #3 | 169 |
| - grey literature from the National Institute for Health Research Centre |  |
| #1 "Breast Neoplasm" OR "Neoplasm, Breast" OR "Breast Tumors" OR "Breast Tumor" OR "Tumor, Breast" OR "Tumors, Breast" OR "Neoplasms, Breast" OR "Breast Cancer" OR "Cancer, Breast" OR "Mammary Cancer" OR "Cancer, Mammary" OR "Cancers, Mammary" OR "Mammary Cancers" OR "Malignant Neoplasm of Breast" OR "Breast Malignant Neoplasm" OR "Breast Malignant Neoplasms" OR "Malignant Tumor of Breast" OR "Breast Malignant Tumor" OR "Breast Malignant Tumors" OR "Cancer of Breast" OR "Cancer of the Breast" OR "Mammary Carcinoma, Human" OR "Carcinoma, Human Mammary" OR "Carcinomas, Human Mammary" OR "Human Mammary Carcinomas" OR "Mammary Carcinomas, Human" OR "Human Mammary Carcinoma" OR "Mammary Neoplasms, Human" OR "Human Mammary Neoplasm" OR "Human Mammary Neoplasms" OR "Neoplasm, Human Mammary" OR "Neoplasms, Human Mammary" OR "Mammary Neoplasm, Human" OR "Breast Carcinoma" OR "Breast Carcinomas" OR "Carcinoma, Breast" OR "Carcinomas, Breast"  #2 "Social Stigma" OR "Social Stigmas" OR "Stigmas, Social" OR "Stigma, Social" OR "Stigma" OR "internalized stigma" OR "externalized stigma" OR "selfstigma" OR "shame"  #3 "influence factors" OR "related factors" OR "risk factors" OR "predictive factors" OR "current situation" OR "investigation" OR "relevance"  #4 #1 AND #2 AND #3 | 0 |
