# Supplementary material for: Factors influencing stigma in Chinese postoperative breast cancer patients: a systematic review and meta-analysis
Source: Front Med (Lausanne). 2025 Oct 9;12:1681487. doi: 10.3389/fmed.2025.1681487 (PMC12546193; doi:10.3389/fmed.2025.1681487)
Supplement: Supplementary file 2 [file Table_2.docx]

**Supplementary Material 2**——Excluded studies and reasons

***- Failed to extract valid data (n=20)***

1. 年轻乳腺癌患者病耻感现状及其影响因素分析
2. 乳腺癌患者术后病耻感及影响因素研究
3. 乳腺癌术后患者病耻感影响因素分析及与自尊、生活质量和心理社会适应能力的关系
4. 年轻乳腺癌患者病耻感与自尊水平的相关性
5. 病耻感在青年乳腺癌患者领悟社会支持与社会疏离间的中介作用
6. 自我表露在乳腺癌幸存者病耻感和孤独感间的中介效应
7. 孤独感和社会支持在农村乳腺癌幸存者病耻感与社交回避间的中介调节作用
8. 桂北地区乳腺癌患者术后生命质量现状及其与病耻感的相关性分析
9. 乳腺癌改良根治术后病耻感与社会支持、家庭关怀度相关性分析
10. 乳腺癌患者病耻感、心理弹性水平及其对社交焦虑的影响
11. 山东省某两所医院乳腺癌术后患者病耻感水平与生命质量的相关性研究
12. 乳腺癌病人术后心理弹性在家庭复原力与病耻感间的中介效应
13. 应对方式在乳腺癌改良根治术患者病耻感与生活质量间的中介效应分析
14. 年轻乳腺癌术后患者病耻感的调查研究
15. 乳腺癌患者家庭回避癌症沟通、婚姻质量与病耻感的相关性研究
16. 乳腺癌患者病耻感的影响因素研究
17. 社会关系质量在乳腺癌幸存者病耻感与社交回避及苦恼间的中介效应
18. 乳腺癌病人社交焦虑和病耻感在家庭关怀度与孤独感间的中介作用
19. 乳腺癌改良根治术后患者病耻感的调查研究
20. Correlates of body image difficulties following breast cancer surgery

***- Stigma was not a major outcome Measure (n=8)***

1. The contribution of self-esteem and self-concept in psychological distress in women at risk of hereditary breast cancer
2. Relationship between cancer stigma, social support, coping strategies and psychosocial adjustment among breast cancer survivors
3. The associations of self-stigma, social constraints, and sleep among Chinese American breast cancer survivors
4. 乳腺癌术后患者出院准备度与病耻感、应对方式的相关性研究
5. 乳腺癌幸存者社会疏离与病耻感、应对万式的关系研究
6. 病耻感在女性乳腺癌术后患者身体意象与性功能障碍中的中介效应研究
7. 乳腺癌生存者重返工作后病耻感对工作行为的影响
8. 乳腺癌患者术后社交回避与苦恼现状及其影响因素分析

***- unreported influencing factors of stigma (n=7)***

1. 病耻感在青年乳腺癌患者领悟社会支持与社会疏离间的中介作用
2. 年轻乳腺癌术后患者病耻感的调查研究
3. 青年乳腺癌术后患者病耻感及应对方式与心理社会适应的相关性研究
4. 乳腺癌病人病耻感及家庭功能与生命质量的关系模型研究
5. 乳腺癌病人术后病耻感与社会支持的相关性研究
6. 乳腺癌术后患者病耻感与生活质量的相关性分析
7. 晚期乳腺癌患者病耻感与生命质量的相关性研究

***- Research subject was not postoperative or Chinese patients (n=3)***

1. Önegyüttérzés és stigmatizáció vizsgálata emlődaganatos nők körében
2. Ambivalence over Emotional Expression and Intrusive Thoughts as Moderators of the link between Self-Stigma and Depressive Symptoms among Chinese American Breast Cancer Survivors
3. Stigma Perceived by Women Following Surgery for Breast Cancer

***- No full-text literature found (n=1)***

1. Does perceived stigma or shame affect stage of presentation in Iraqi Kurdish women diagnosed with breast cancer?
